# Effects of feeding an inoculated corn silage with or without a direct-fed microbial on dry matter intake, milk production, and nutrient digestibility of high-producing lactating Holstein cows

**DOI:** 10.1093/tas/txae010

**Published:** 2024-01-14

**Authors:** Ivonne Kok, Giuseppe Copani, Keith A Bryan, Kristian L M Witt, Wilfried M van Straalen, Rafael C do Amaral, Bruno I Cappellozza

**Affiliations:** Schothorst Feed Research, 8218 NALelystad, The Netherlands; Animal and Plant Health & Nutrition, Chr. Hansen A/S, Hørsholm 2970, Denmark; Animal and Plant Health & Nutrition, Chr. Hansen, Inc., Milwaukee, WI 53214, USA; Animal and Plant Health & Nutrition, Chr. Hansen A/S, Hørsholm 2970, Denmark; Schothorst Feed Research, 8218 NALelystad, The Netherlands; Animal and Plant Health & Nutrition, Chr. Hansen Indústria e Comércio, Valinhos, SP 13278-327, Brazil; Animal and Plant Health & Nutrition, Chr. Hansen A/S, Hørsholm 2970, Denmark

**Keywords:** bacterial silage inoculant, direct-fed microbial, milk production efficiency, milk yield, nutrient digestibility

## Abstract

This study evaluated the effects of inoculating corn silage and/or feeding a direct-fed microbial (PRO) on performance and nutrient digestibility of lactating dairy cows. At harvesting, corn silage was treated either with water (culated or not [CON]) or *Lactococcus lactis* and *Lentilactobacillus buchneri* (INC; SiloSolve FC) at 1.5 × 10^5^ cfu/g of corn silage. Ten mini silos and one farm-scale silo bunker per treatment were prepared for the laboratory and the lactating dairy cow trial, respectively. Five mini silos per treatment were opened on days 2 or 90 post-ensiling for pH measurement, as well as chemical analysis and aerobic stability, respectively. The farm-scale silo bunkers were opened 77 d post-ensiling for the beginning of the lactating cow trial. Eighty lactating Holstein cows were assigned in a 2 × 2 factorial design to: (1) CON silage without PRO (CON-CON; *n* = 20), (2) CON silage with PRO at 14 g/head/d (CON-PRO; *n* = 20), (3) INC silage without PRO (INC-CON; *n* = 20), and (4) INC silage with PRO at 14 g/head/d (INC-PRO; *n* = 20). Concurrently with the feeding trial, eight cows per treatment were chosen for nutrient digestibility. The pH of the corn silage was not affected by the silage inoculant (*P* ≥ 0.29), but INC yielded greater concentration of acetic acid and longer aerobic stability (*P* < 0.01). Dairy cows fed INC had a lower mean total dry matter intake (DMI), milk protein content, and somatic cell counts vs. CON (*P* ≤ 0.02). On the other hand, milk and fat- and protein-corrected milk (FPCM) production efficiency, milk urea-N, DM, crude protein, and starch digestibility were greater for INC-fed cows (*P* ≤ 0.03). Feeding direct-fed microbials (DFM) improved mean body weight, milk yield, and FPCM, as well as milk protein and lactose yield (*P* ≤ 0.05), but reduced milk fat and protein content (*P* = 0.02). A silage inoculant × DFM interaction was observed for milk production efficiency, milk protein and lactose content, and somatic cell count (*P* ≤ 0.05). Dairy cows fed INC-CON had a greater milk production efficiency and milk lactose content (*P* ≤ 0.04), but INC-PRO had lower milk protein content and SCC (*P* ≤ 0.03). In summary, inoculating *L. lactis* and *L. buchneri* increased acetic acid content and aerobic stability of corn silage, reduced DMI, but improved milk production efficiency and nutrient digestibility of lactating Holstein dairy cows. On the other hand, feeding PRO improved milk, protein, and lactose yield. Additionally, combining the feeding of an inoculated corn silage with PRO reduced milk somatic cell count.

## Introduction

Lactic acid bacteria have been used for decades as silage inoculants to improve silage fermentation profile, to control the growth and development of yeast and molds (Weinberg and Muck, 1996), and to reduce nutrient losses during the fermentation process (Muck, 2013). Homofermentative bacterial inoculants, such as *Lactococcus lactis*, promote acidification of the feedstuff during fermentation due to their ability to produce lactic acid and reduce the pH of the ensiled material (Kung et al., 2003; White, 2007; Hindrichsen et al., 2012). The faster the pH falls, the sooner the undesired side effects of the fermentation process may be halted, including proteolysis and sugar consumption (Kung et al., 2003). Conversely, heterofermentative bacterial inoculants are applied to increase the production of acetate, which provides antifungal properties and increases aerobic stability of silages (Schmidt and Kung, 2010; Comino et al., 2014). The variation in the effectiveness of heterofermentative bacterial inoculants may be attributed to differences in strains, crop types, and ensiling conditions (Muck and Kung, 1997).

*Lentilactobacillus* (formerly known as *Lactobacillus*) *buchneri* is the most common and most studied heterofermentative bacteria used in silage inoculants. One may speculate that the combination of *L. lactis* and *L. buchneri* would improve aerobic stability of the silage due to its effective antimicrobial and antifungal activity. Recently, Saylor et al. (2020a) reported longer aerobic stability, greater soluble crude protein (CP) proportion, and greater in situ ruminal starch degradation when *L. lactis* and *L. buchneri* were applied in high-moisture corn silage. The effects of inoculated silages on animal performance have been reported by others (Oliveira et al., 2017; Queiroz et al., 2017) with slight positive effects on cow dry matter intake (DMI) and milk yield, but no improvements on total-tract nutrient digestibility (Oliveira et al., 2017). The positive effect of feeding inoculated silages on DMI is often attributed to an increased silage palatability and reduced concentration of undesired compounds that can depress DMI (Daniel et al., 2013; Gerlach et al., 2021). However, no studies have evaluated the effects of feeding a corn silage treated with *L. lactis* and *L. buchneri* on performance of lactating dairy cows.

Direct-fed microbials (DFM) have been fed to dairy cattle to support rumen and lower gastrointestinal tract (GIT) health, increase nutrient digestibility, and promote milk production (Seo et al., 2010; McAllister et al., 2011). Among the microorganisms used as DFM for cattle, *Enterococcus* spp. and live yeast (Shridhar et al., 2022; Salah et al., 2023) have been widely used, alone or in combination, with successful results on nutrient digestibility, DMI, and milk yield of transition dairy cows, as well as maintaining a less acidic rumen pH in subacute rumen acidosis (SARA)-challenged dairy cows (Nocek et al., 2003; Nocek and Kautz, 2006; Chiquette et al., 2015). Nonetheless, the effects of feeding a combination of bacteria and yeast on performance and nutrient digestibility of dairy cows fed a bacterial inoculated-based partial mixed ration (PMR) is lacking and warrants further investigation. Based on this rationale, we hypothesized that (1) adding a combination of *L. lactis* and *L. buchneri* (SiloSolve FC; Chr. Hansen A/S) to corn silage would alter its acid profile and increase its aerobic stability, and (2) feeding DFM (Probios Complete) to cows receiving a bacterial inoculated corn silage would have an additive effect on production performance when compared with feeding inoculated silage or feeding DFM alone. Therefore, our objective was to evaluate the effects of inoculating corn silage with *L. lactis* and *L. buchneri* (SiloSolve FC) on its acid profile and aerobic stability, as well as whether adding DFM (Probios Complete) to the inoculated silage would improve nutrient digestibility and productive performance of lactating Holstein dairy cows.

## Materials and Methods

### Mini Silos Trial

All experiments were conducted at the Schothorst Feed Research facility (Lelystad, The Netherlands; 52°31’6’’N, 5°28’17’’E, and an elevation of −2.9 m). Whole crop corn hybrid LG30.225 (Limagrain, Rilland, The Netherlands) was harvested in October 2015 (DM content of 33%) using a forage harvester (FX-50; New Holland Agriculture, Turin, Italy) that chopped the material to a pre-planned length of 0.6 cm of particle size. From each wagon of untreated corn silage for the feeding and digestibility experiment, a grab sample was taken. Grab samples were collected and mixed thoroughly to provide a representative pooled sample of ~300 kg for the mini silo trial. Mini-silos (*n* = 20; capacity of 10 L each) were filled with equal amounts of chopped corn silage and randomly received one of the following treatments: (1) water addition only (culated or not [CON]; *n* = 10) or (2) addition of 1.5 × 10^5^ cfu/g of corn silage of an inoculant containing *L. lactis* (7.5 × 10^4^ cfu/g) and *L. buchneri* (7.5 × 10^4^ cfu/g); INC; *n* = 10; (SiloSolve FC; Chr. Hansen A/S, Hørsholm, Denmark). The bacterial inoculant was prepared by mixing the commercial product in water and treatments (CON and INC) were applied at a rate of 3 mL of solution/kg of corn silage. The treatments were applied evenly to each silo and thoroughly mixed in a plastic bag before packing in the mini silo container, whereas fresh chopped corn silage from each treatment was sampled in duplicate for further laboratory analysis.

The forage was packed in mini silos by a hydraulic press to achieve a packing density of 750 kg of fresh material/m^3^, and mini silos were closed and sealed immediately after filling. Throughout the experimental period, the silos were stored at room temperature (20 ± 5 °C). Ten mini silos (*n* = 5/treatment) were opened 2 d after their preparation for pH measurement with a digital pH reader, whereas the remaining silos were opened after 90 d for chemical composition and aerobic stability determination, as described below. Immediately after opening the mini silo on day 90, a 1 kg sample was collected from each mini silo for determination of aerobic stability following the methodology previously described by Honig (1990). Thermocouple wires were placed at the center of each silage sample and were connected to a data logger (Testo Saveris 21CFR-11 Software; Testo BV, Almere, The Netherlands) that recorded temperature every 10 min. Two thermocouple wires were attached to the facilities in the open air to continuously measure environmental temperature. Aerobic stability measurement continued for 2 wk post-mini silo opening and was defined as the number of hours before the silage temperature exceeded ambient temperature by 3 °C (Honig, 1990).

An additional corn silage sample was taken in duplicate on days 0 and 90 and stored at -20 °C for further chemical analysis. Dry matter content of the samples was determined in a forced-air oven (70 °C for 16 h). After drying, samples were ground through a 1-mm screen (Peppink-200 AN-C mill; Peppink Mills BV, Overijssel, The Netherlands) analyzed by near-infrared spectroscopy (NIRS; Quant FT-NIR analyzer; Q-Interline A/S, Tølløse, Denmark) for ash, ammonia-N, CP, crude fat, neutral detergent fiber (NDF), acid detergent fiber (ADF), acid detergent lignin (ADL), sugar, starch, pH, and digestibility coefficients for organic matter and NDF. Table 1 reports the nutritional profile of the corn silage samples obtained on day 0 of the study. Samples of the corn silage collected on day 90 were also analyzed for lactic, acetic, butyric, propionic, and formic acids by HPLC following the methodology described by Canale et al. (1984).

**Table 1.** Chemical composition of fresh corn silage samples obtained at harvesting (day 0) and immediately before being ensiled in the mini silos^1^^,^^2^

Item	Treatments		SEM
	CON	INC	
Dry matter (DM), g/kg	327	332	4.7
Crude protein (CP), g/kg DM	74	70	1.8
Neutral detergent fiber (NDF), g/kg DM	361	380	1.8
Acid detergent fiber (ADF), g/kg DM	184	198	2.7
Acid detergent lignin (ADL), g/kg DM	13	13	0.0
Sugar, g/kg DM	58	58	1.1
Starch, g/kg DM	375	359	11.3
Digestibility coefficient for organic matter (DC-OM)^3^, %	79.9	80.4	0.04
Digestibility coefficient for NDF (DC-NDF)^3^, %	65.6	64.0	0.06

^1^Treatments included the addition or not (CON) of 1.5 × 10^5^ cfu/g of corn silage of *L. lactis* and *Lentilactobacillus* (formerly known as *Lactobacillus*) *buchneri* (INC; SiloSolve FC; Chr. Hansen A/S, Hørsholm, Denmark).

^2^Samples were taken in duplicates.

^3^Determined according to the near-infrared spectroscopy (NIRS).

### Lactation and Digestibility Trial

All animal procedures followed the recommendations of the Guide for the Care and Use of Agricultural Animals in Agricultural Research and Teaching (FASS, 2010). All animals used herein were cared for in accordance with acceptable practices and all procedures related to the Dutch Law on Animal Experiments were evaluated and approved by the Central Authority for Scientific Procedures on Animals (CCD, Den Haag, The Netherlands; approval # AVD2460022015172).

The corn that was harvested for the mini silo trial was also ensiled in farm-scale bunker silos (20.0 × 7.0 × 1.7 m) for a subsequent in vivo lactation and digestibility study. Half of the corn crop was untreated (CON) while the other half was treated with the same bacterial silage inoculant reported for the mini silo trial and at the same dosage (1.5 × 10^5^ cfu/g of corn silage; INC). The inoculant was applied in water (0.8 L/metric ton) by a mounted sprayer (BalFor A9 V-OR/500EM; Ballario & Forestello, Manta, IT) that was installed in the forage harvester. Of ~8 wk post-ensiling, corn silage samples were taken from both treatments for PMR formulation, whereas the full opening of the silo for the trial occurred 3 wk later (77 d).

The lactation experiment used 80 lactating primiparous (*n* = 24) and multiparous (*n* = 56) Holstein cows (114 d in milk; range from 55 to 184 d) with an average fat- and protein-corrected milk (FPCM) yield of 38.4 ± 8.49 kg. Three weeks before the beginning of treatment administration, all cows were fed the same basal diet that did not contain any silage inoculant and/or DFM. On day 0 of the study, cows were blocked by parity, days in milk, and FPCM, and within blocks randomly assigned to the following treatments in a 2 × 2 factorial design: (1) CON corn silage without the addition of DFM (CON-CON; *n* = 20), (2) CON corn silage with the addition of 14 g/head per day of a DFM containing three strains of *E. faecium* (3.53 × 10^8^ CFU/g), one strain of *S. cerevisiae*, and one strain of Torula (*Cyberlindnera jadinii*) yeast (3.53 × 10^11^ CFU/g; Probios Complete; Chr. Hansen, Inc., Milwaukee, WI, USA; CON-PRO; *n* = 20), (3) INC corn silage without the addition of DFM (INC-CON; *n* = 20), and (4) INC corn silage with the addition of 14 g/head/d of DFM (INC-PRO; *n* = 20). The experimental period lasted 13 wk and the DFM was mixed daily with ground corn and wheat middlings to ensure a supply of 14 g/cow/d, whereas the CON group received the same amount of the ground corn:wheat middling mixture without DFM twice a day in individual concentrate feeders.

The diet offered throughout the experimental period was formulated following the guidelines by CVB (2022). The PMR offered to cows contained 68% corn silage (CON or INC), 28% grass silage, and 4% soybean meal (DM basis). The PMR was fed twice daily in amounts to ensure ad libitum consumption, whereas feed refusals were weighed and recorded daily for determination of individual cow DMI. A commercial concentrate supplement (ABZ Diervoeding, Nijkerk, The Netherlands) containing (as-fed basis) 20.0% corn gluten feed, 19.0% palm kernel expeller, 11.3% soybean meal, 11.0% lupins, 9.0% beet pulp, 7.3% ground corn, 5.0% rapeseed meal, 4.0% sugarcane molasses, 4.0% formaldehyde-treated soybean meal, 3.6% soybean hulls, 3.0% peas, 2.6% mineral-vitamin premix, and 0.2% soybean oil was offered at a rate of 3.0 kg/cow/d to meet the nutrient requirements of lactating dairy cows producing at least 38.0 kg of milk daily. The nutritional profile of the PMR and concentrate supplement offered during the entire experimental period is reported in Table 2. Throughout the experimental period, the grass silage, soybean meal, and concentrate feed offered to all cows were the same, whereas the corn silage was taken from CON or INC batches. For the corn silage handling and management, the CON diet was always prepared and mixed prior to the INC one, so that the risk of cross-contamination would be alleviated among cows from the different treatment groups.

**Table 2.** Composition and nutritional profile of the ingredients used in the partial mixed ration (PMR) and the concentrate fed to the dairy cows in the lactation trial

Item	Concentrate	Grass silage	Corn silage^1^^,^^2^		Diets	
			CON	INC	CON	INC
Dry matter, g/kg	896	398	333	320	443	436
CP, g/kg DM	246	152	74	74	165	165
Ammonia-N, % CP	—	10	9	9	—	—
NDF, g/kg DM	336	481	350	357	352	354
ADF, g/kg DM	186	276	193	207	197	203
ADL, g/kg DM	31	19	15	17	18	19
Starch, g/kg DM	108	—	374	360	210	199
Crude fat, g/kg DM	49	39	31	31	35	35
Ash, g/kg DM	78	100	41	46	64	67
Sugar, g/kg DM	66	90	12	12	45	46
Acetic acid, g/kg DM	—	15	13	15	—	—
Lactic acid, g/kg DM	—	41	48	53	—	—
DC-OM^3^, %	—	78.1	78.3	76.5	—	—
DC-NDF^3^, %	—	74.7	54.9	53.5	—	—
VEM^4^,/kg DM	1,088	923	1,008	974	1,028	1,009
TMP^5^, g/kg DM	134	67	60	58	95	95
RPB^6^, g/kg DM	57	27	−48	−48	15	16

^1^Treatments included the addition or not (CON) of 1.5 × 10^5^ cfu/g of corn silage of *L. lactis* and *Lentilactobacillus* (formerly known as *Lactobacillus*) *buchneri* (INC; SiloSolve FC; Chr. Hansen A/S, Hørsholm, Denmark).

^2^Corn silage composition based on samples pooled during the experimental period.

^3^Determined according to the near-infrared spectroscopy (NIRS).

^4^Feeding Unit Milk, NE_L_ relative to barley (CVB, The Netherlands).

^5^Total metabolizable protein (E-dairy, Schothorst Feed Research, Lelystad, The Netherlands).

^6^Rumen protein balance (E-dairy, Schothorst Feed Research, Lelystad, The Netherlands).

*Sampling*. All cows were housed in a loose, free-stall housing system containing individual feeding bunkers with a transponder-operated system (Calan, American Calan; Northwood, NH, USA) for measurement of individual feed intake. Cows were milked twice daily and the milk was sampled for milk composition twice per week by compositing a sample from the morning and evening milking. Milk samples were individually analyzed for fat, protein, lactose, and urea using the NIRS methodology, as well as somatic cell counts by fluorescence microscopy (Qlip, Zutphen, The Netherlands). Based on the milk composition results, the FPCM yield was calculated by averaging the weekly milk production and composition, and using the following equation:

FPCM (kg/d) = MY × [0.337 + 0.116 × MFC (%) + 0.06 × MPC (%)], where MY = milk yield in kg/d, MFC = milk fat content in %, and MPC = milk protein content in %.

Individual cow body weight (BW) was recorded twice daily via an automatic digital scale located at the end of the milking parlor, whereas cow BCS was determined in weeks 0, 4, 9, and 13 of the study according to methodology described by Edmonson et al. (1989). Silage (corn and grass) samples were collected every week and composited for NIRS analyses at the end of the experiment (week 13). Concentrates were sampled per batch being produced during the experiment and were analyzed by wet chemistry for nutrient composition. Dry matter content was determined after drying the samples in an oven at 103 °C for 12 h (ISO 6492., 1999a). Combustion for 3 h in a muffle at 550 °C was used to determine ash content (ISO 2002, 5984), whereas nitrogen (N) was analyzed according to the Dumas method (ISO 16634-1, 2008b) and CP calculated as *N* × 6.25. Starch content was determined by using an enzymatic extraction of sugars in an ethanol-based solution (ISO 15914, 4, 2004). Crude fat was analyzed by ether extraction after acid hydrolysis (ISO 6496., 1999b). NDF was determined according to the method of Van Soest (ISO 16472, 2, 2006) after pretreatment with *α*-amylase, whereas ADF and ADL were determined according to the methodology developed by van Soest (ISO 13096, 2008a).

*Apparent nutrient digestibility*. For measurement of total-tract apparent nutrient digestibility, 8 blocks of cows from the previously described lactation trial were fed titanium dioxide (TiO_2_; # 1.00808; Merck Life Science, Darmstadt, Germany) during weeks 3 and 16 of the experimental period. The TiO_2_ was added at 1.5% of the concentrate feed that was fed to the cows on a daily basis (3 kg of concentrate/cow/d). During week 4, the feed ingredients were sampled daily for 5 d and fecal samples were manually collected twice daily (between 0800 to 1000 hours and 1330 to 1530 hours) for 4 d. Fecal samples were composited per cow, dried for DM determination, freeze-dried, and analyzed by wet chemistry methods as described above for CP, starch, and NDF. Titanium was analyzed by spectrophotometric method after combustion and destruction with sulfuric acid (Short et al., 1996). Feed samples collected during this period were composited per type and analyzed by wet chemical methods for CP, starch, and NDF, whereas concentrates were also analyzed for Ti. Total-tract apparent nutrient digestibility was calculated via theoretical fecal excretion assuming that all ingested Ti was excreted in the feces, satisfying the assumption of an external marker.

### Statistical Analyses

All data were analyzed with SAS Statistical Software (version 9.4; SAS Inst. Inc., Cary, NC, USA). The mini silo experiment was analyzed as a complete randomized design having five replications per treatment on days 2 and 90 post-mini silo preparation. The mini silo was used as the random variable, whereas treatment was included as the fixed effect. In the performance and digestibility experiment, all data were analyzed using cow as the experimental unit in a 2 × 2 factorial design, with factor 1 being the inoculation or not of the corn silage (CON or INC) and factor 2 being the feeding or not of the DFM (CON or PRO). Factors 1 and 2 were considered the fixed effects, whereas block was used as the random variable, and values obtained before treatment administration were used as covariates for DMI, milk yield, milk composition, milk production efficiency, and total-tract apparent nutrient digestibility.

For the analyses conducted herein, all data were reported as least square means and covariately adjusted to the values obtained prior to treatment administration in the lactation trial (DMI, milk yield, milk composition, and milk production efficiency). Significance was set at *P* ≤ 0.05, tendencies denoted if 0.05 < *P* ≤ 0.10, and results were reported according to the main effects if no interactions were significant (*P* ≤ 0.05).

## Results

### Mini Silos Trial

As reported in Table 1, the chemical composition of the fresh corn harvested and used for the mini silo trial was similar between treatments.

No treatment effects were observed on pH measured either on days 2 or 90 post-mini silo preparation (*P* ≥ 0.29; Table 3). Moreover, no differences were observed in lactic acid, overall nutrient composition, and calculated nutritional value of corn silages inoculated or not with a bacterial ensiling agent (*P* ≥ 0.25). On the other hand, INC corn silages had a greater concentration of acetic acid (*P* < 0.01) and longer aerobic stability (*P* < 0.01), while starch content also tended to be slightly higher in INC vs. CON (*P* = 0.08; Table 3).

**Table 3.** Effects of adding a bacterial silage inoculant on fermentation profile, nutrient composition, and calculated nutritional value of corn silage ensiled in mini silos for 90 d^1^^,^^2^

Item	CON	INC	SEM	*P* =
*Fermentation profile*				
pH				
Day 2	4.2	4.2	0.01	0.29
Day 90	3.9	3.9	0.02	1.00
Lactic acid, g/kg DM	64	63	0.57	0.25
Acetic acid, g/kg DM	14	19	0.37	<0.01
Propionic acid, g/kg DM	<0.5	<0.5	—	—
Formic acid, g/kg DM	<0.5	<0.5	—	—
Butyric acid, g/kg DM	<0.5	<0.5	—	—
Nitrate, g/kg DM	<0.2	<0.2	—	—
Aerobic stability, h	69	219	31.2	<0.01
*Nutrient composition*				
DM, g/kg	314	313	1.5	0.53
CP, g/kg DM	71	72	0.7	0.28
Ammonia-N, % CP	8.6	8.4	0.25	0.58
NDF, g/kg DM	335	330	6.1	0.55
ADF, g/kg DM	184	185	3.4	0.85
ADL, g/kg DM	13	12	0.2	0.25
Sugar, g/kg DM	12	12	0.0	1.00
Starch, g/kg DM	369	384	5.1	0.08
DC-OM^3^, %	80.2	80.0	0.2	0.46
DC-NDF^3^, %	59.8	59.9	0.5	0.92

^1^Treatments included the addition or not (CON) of 1.5 × 10^5^ cfu/g of corn silage of *L. lactis* and *Lentilactobacillus* (formerly known as *Lactobacillus*) *buchneri* (INC; SiloSolve FC; Chr. Hansen A/S, Hørsholm, Denmark).

^2^Five mini silos per treatment were evaluated on day 90 post-ensiling.

^3^Determined according to the near-infrared spectroscopy (NIRS).

**Figure 1.**
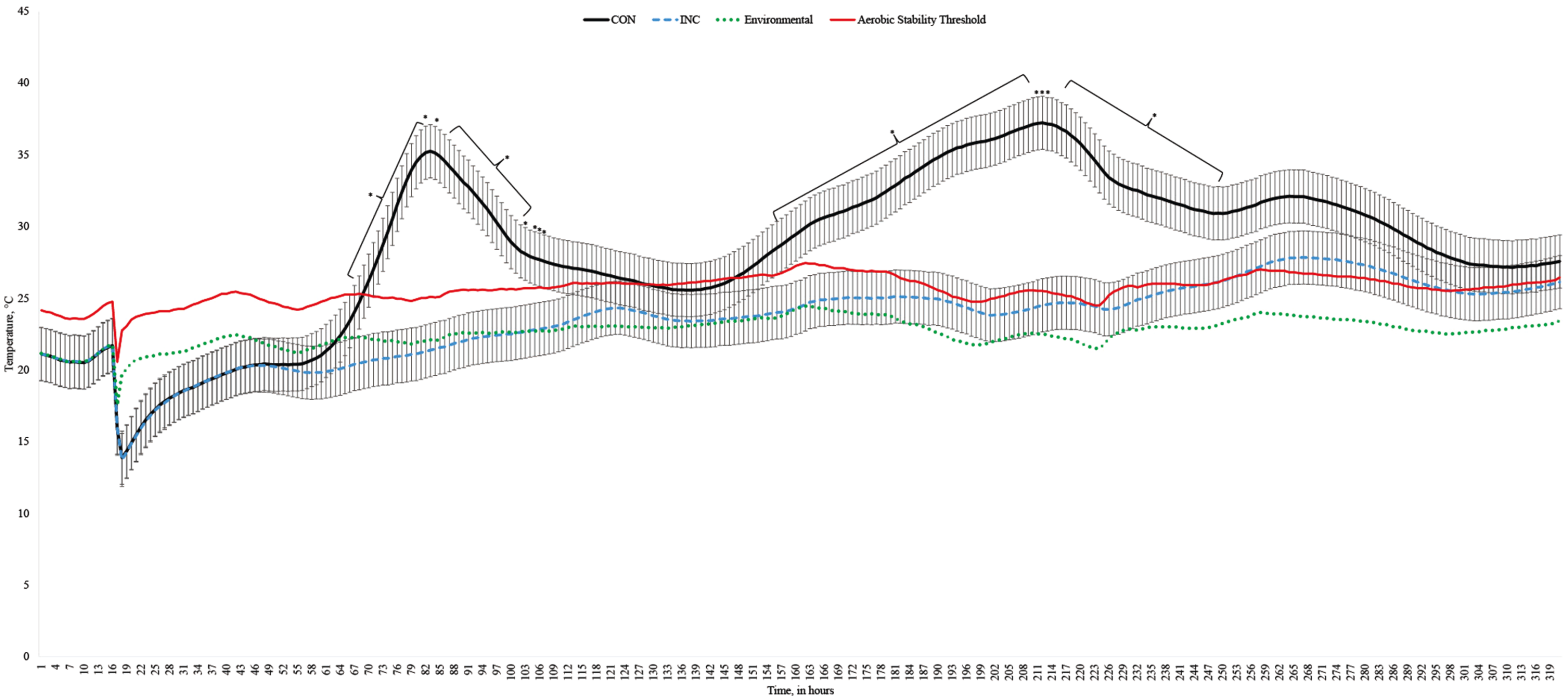
Temperature of corn silages inoculated or not (CON) with 1.5 × 105 cfu of *Lactococcus lactis* and *Lentilactobacillus* (formerly known as *Lactobacillus*) buchneri per g of corn silage (INC; SiloSolve FC; Chr. Hansen A/S, Hørsholm, Denmark). Corn silage was ensiled for 90 d. A treatment × hour interaction was observed (*P* < 0.0001; SEM = 1.85). * denotes differences at *P* ≤ 0.05. level.

A treatment × hour interaction was observed for corn silage temperature after mini silo opening on day 90 for aerobic exposure (*P* < 0.001; Figure 1). Inoculating the corn silage with *L. lactis* and *L. buchneri* resulted in lower temperatures from 68 to 111 h (*P* ≤ 0.05) and from 154 to 258 h (*P* ≤ 0.05; Figure 1). The environmental temperature ranged from 17.6 to 24.5 °C, whereas it ranged from 13.9 to 37.2 °C and from 13.7 to 27.8 °C for CON and INC, respectively.

### Lactation and Digestibility Trial

For all analyses performed here, no silage inoculant × week, direct-fed microbial × week, or silage inoculant × direct-fed microbial × week interactions were observed (*P* ≥ 0.65); hence, only the results from the main effects and silage inoculant × direct-fed microbial results will be reported. Moreover, all variables were significant covariates (*P* ≤ 0.01) but did not differ due to the main effects of the bacterial silage inoculant (*P* ≥ 0.24), DFM (*P* ≥ 0.09), or the resulting interaction (*P* ≥ 0.10), with FPCM being the only exception (*P* = 0.05; data not shown).

#### Silage inoculant effect.

Dairy cows fed INC had a lower mean total DMI, milk protein percentage, and somatic cell counts when compared with CON cohorts (*P* ≤ 0.02; Table 4). On the other hand, milk and FPCM production efficiency, as well as milk urea-N was greater for cows fed INC silage (*P* ≤ 0.0001), but no further silage inoculant effects were observed on average BW, milk yield, milk composition yield, and fat and lactose milk content (*P* ≥ 0.11; Table 4).

**Table 4.** Effects of adding or not (CON) a bacterial silage inoculant (INC) on corn silage and/or feeding a direct-fed microbial (DFM) on performance of lactating dairy cows^1^^,^^2^

Item	Silage inoculant		DFM		SEM	*P*		
	CON	INC	CON	PRO		Inoculant	DFM	Inoculant × DFM
Body weight, kg	662.7	662.1	659.4	665.4	3.37	0.79	<0.01	0.86
Dry matter intake, kg/d	23.2	22.7	22.9	23.0	0.24	<0.0001	0.14	0.47
Milk yield, kg/d	35.1	35.0	34.7	35.4	0.33	0.52	<0.01	0.85
Fat- and protein-corrected milk (FPCM), kg/d	36.4	36.1	36.0	36.4	0.32	0.19	0.05	0.80
Milk production efficiency, kg/kg	1.55	1.58	1.56	1.57	0.009	<0.0001	0.58	0.05
FPCM efficiency, kg/kg	1.55	1.58	1.56	1.57	0.009	0.0001	0.73	0.34
Milk composition, kg/d								
Fat	1.47	1.45	1.46	1.46	0.014	0.15	0.63	0.93
Protein	1.25	1.24	1.24	1.26	0.013	0.11	<0.01	0.23
Lactose	1.61	1.60	1.59	1.62	0.016	0.55	<0.01	0.73
Milk composition, %								
Fat	4.23	4.21	4.24	4.19	0.032	0.44	0.02	0.45
Protein	3.60	3.57	3.60	3.57	0.021	0.02	0.02	0.03
Lactose	4.57	4.58	4.57	4.58	0.008	0.24	0.39	<0.01
Milk urea-N, mg/dL	20.9	21.9	21.3	21.4	0.31	<0.0001	0.60	0.19
Somatic cell counts, log_10_	1.79	1.72	1.77	1.75	0.024	<0.0001	0.35	0.03

^1^Silage inoculant treatments included the addition or not (CON) of 1.5 × 10^5^ cfu/g of corn silage of *L. lactis* and *Lentilactobacillus* (formerly known as *Lactobacillus*) *buchneri* (INC; SiloSolve FC; Chr. Hansen A/S, Hørsholm, Denmark).

^2^Lactating dairy cows also received or not 14 g/head/d of a direct-fed microbial (DFM) containing three strains of *Enterococcus faecium*, one strain of *Saccharomyces cerevisiae*, and one strain of Torula (Probios Complete; Chr. Hansen, Inc., Milwaukee, WI, USA) for 13 wk.

Regarding apparent nutrient digestibility, feeding INC improved DM, CP, and starch digestibility (*P* ≤ 0.03), while also tending to increase NDF digestibility when compared with CON cows (*P* = 0.10; Table 5).

**Table 5.** Effects of adding or not (CON) a bacterial silage inoculant (INC) on corn silage and/or feeding a direct-fed microbial (DFM) on apparent nutrient digestibility of lactating dairy cows^1^^,^^2^

Item	Silage inoculant		DFM		SEM	P =		
	CON	INC	CON	PRO		Inoculant	DFM	Inoculant × DFM
Nutrient digestibility, %								
Dry matter	67.7	69.6	68.9	68.4	0.56	0.02	0.43	0.24
Crude protein	65.8	67.6	66.4	67.0	0.75	0.03	0.42	0.02
Neutral detergent fiber	53.9	56.5	56.0	54.3	1.07	0.10	0.26	0.40
Starch	96.9	97.7	97.3	97.3	0.14	0.0001	0.89	0.68

^1^Silage inoculant treatments included the addition or not (CON) of 1.5 × 10^5^ cfu/g of corn silage of *L. lactis* and *Lentilactobacillus* (formerly known as *Lactobacillus*) *buchneri* (INC; SiloSolve FC; Chr. Hansen A/S, Hørsholm, Denmark).

^2^Lactating dairy cows also received or not 14 g/head/d of a direct-fed microbial (DFM) containing three strains of *Enterococcus faecium*, one strain of *Saccharomyces cerevisiae*, and one strain of Torula (Probios Complete; Chr. Hansen, Inc., Milwaukee, WI, USA) for 13 wk.

#### Direct-fed microbial effect.

Feeding a DFM containing *E. faecium*, *S. cerevisiae*, and Torula yeast improved mean BW of the cows, milk yield, FPCM, as well as milk protein and lactose yield (*P* ≤ 0.05; Table 4). On the other hand, milk fat and protein content were lower for PRO-fed cows (*P* = 0.02). Lastly, no DFM supplementation effects were observed on apparent nutrient digestibility (*P* ≥ 0.26; Table 5).

#### Silage inoculant × DFM interaction.

**Figure 2.**
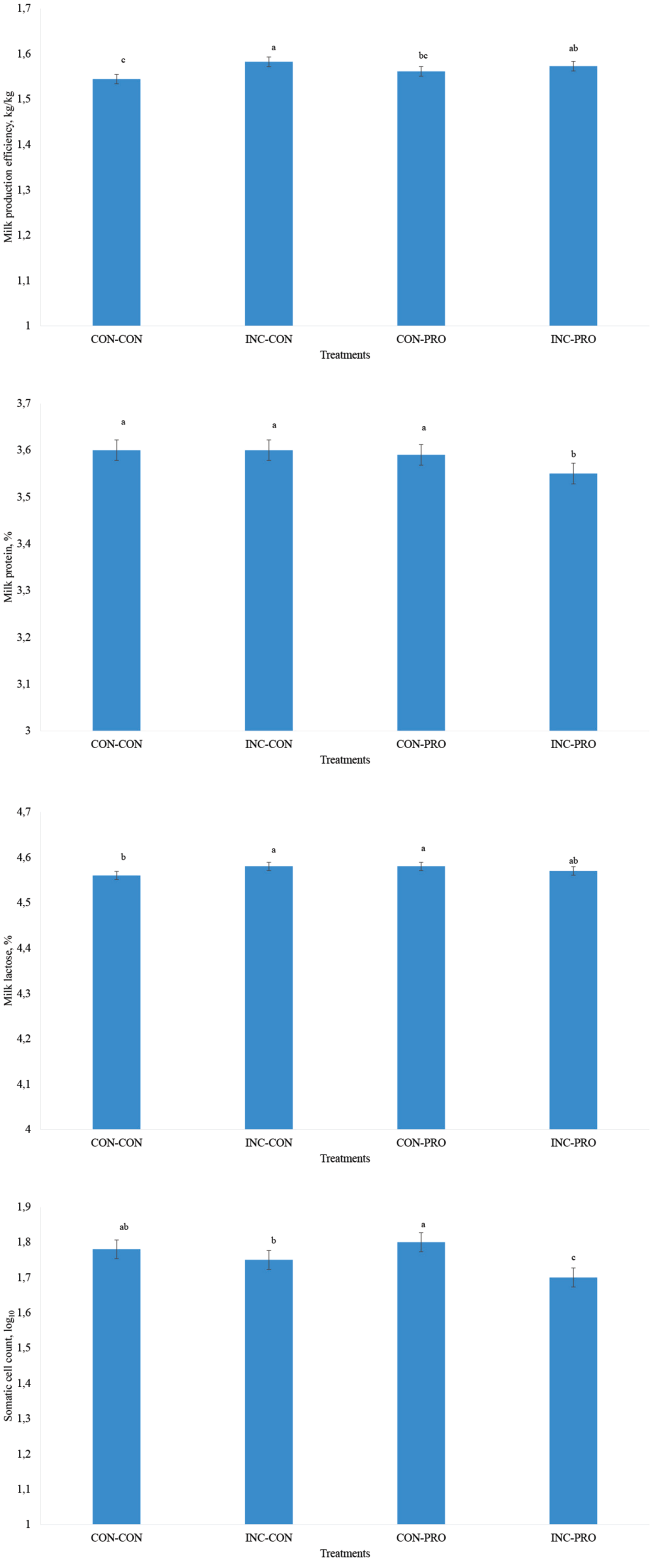
Milk production efficiency (A), milk protein (B) and lactose (C) content, and somatic cell count (D) of lactating dairy cows receiving a corn silage inoculated or not with bacterial inoculants and/or a direct-fed microbial (DFM). A silage inoculant × DFM interaction was observed for all variables (*P* ≤ 0.05). Different letters denote differences at *P* < 0.05.

A silage inoculant × DFM interaction was observed for milk production efficiency, milk protein and lactose content, and somatic cell count (*P* ≤ 0.05; Figure 2A to D). Dairy cows fed INC-CON had a greater milk production efficiency when compared with CON-PRO and CON-CON (*P* ≤ 0.04), but milk production efficiency did not differ from INC-PRO (*P* = 0.34). Moreover, INC-PRO also had a greater milk production efficiency (*P* < 0.01) than CON-CON (Figure 2A).

Milk protein content was lower for INC-PRO when compared with all other treatments (*P* < 0.01), but no differences were observed among CON-CON, INC-CON, and CON-PRO (*P* ≥ 0.84; Figure 2B). On the other hand, milk lactose content was greater for INC-CON and CON-PRO vs. CON-CON (*P* < 0.01), but no differences were observed between the aforementioned treatments and INC-PRO (*P* ≥ 0.14; Figure 2C). Lastly, feeding INC-PRO yielded the lowest SCC, which differed from all other treatments (*P* ≤ 0.03; Figure 2D). Somatic cell count also was lower for INC-CON vs. CON-PRO (*P* = 0.03) and none of the treatments differed from CON-CON (*P* ≥ 0.20; Figure 2D).

No silage inoculant × DFM interaction was observed for DM, NDF, or starch digestibility (*P* ≥ 0.24), but CP digestibility was affected (*P* = 0.02; Table 5). Cows fed CON-CON had lower CP digestibility when compared with other treatments (*P* ≤ 0.03), whereas no further differences were observed among INC-CON, CON-PRO, and INC-PRO (*P* ≥ 0.20; Table 5).

## Discussion

### Effects of Homo- and Heterofermentative Bacterial Inoculant on Corn Silage

Inoculation of corn silage with *L. lactis* and *L. buchneri* improved aerobic stability by 150 h, corresponding to the significant difference in acetic acid concentration (5 g/kg DM or 35.7%). The effectiveness of *L. buchneri* in improving aerobic stability has been reported in the literature (Kleinschmit and Kung, 2006; Schmidt and Kung, 2010; Comino et al., 2014) and explained by the higher concentration of acetic acid in the inoculated silage (Oude Elferink et al., 2001). In corn silage, adding the same *L. lactis* and *L. buchneri* as used herein increased pH, acetic acid, and reduced ammonia-N when compared with an untreated con silage (Saylor et al., 2020b). Predicting the effect of acetic acid content on aerobic stability of silages, Comino et al. (2014) reported that for each 1 g of improvement in silage acetic acid content, aerobic stability increased roughly by 6 h. Others have reported that the increase in the acetic acid content of silages inoculated with ~10^5^ cfu of *L. buchneri*/g of silage was 5 g/kg DM, leading to a 10-h extended aerobic stability of the silage (Kleinschmit and Kung, 2006). Therefore, the rationale and regression analysis results proposed by Comino et al. (2014) partially support, but cannot fully explain, all the improvements in aerobic stability of corn silage inoculated with a combination of *L. lactis* and *L. buchneri*. Recently, Saylor et al. (2020a) reported lower lactic and greater acetic acid concentrations, as well as a prolonged aerobic stability when *L. lactis* and *L. buchneri* were added to high-moisture corn, regardless of grain particle size, ensiled for a maximum period of 28 d. Previous authors suggested that the aerobic stability of corn silage would be maximized at 120 d of fermentation (Daniel et al., 2015), whereas our results demonstrate a significant improvement (+167 h) in aerobic stability of corn silage inoculated with *L. lactis* and *L. buchneri* that was ensiled by ~90 d only.

Our results regarding the effects of *L. lactis* and *L. buchneri* on temperature of the corn silage over a long period of time (up to 320 h) after opening mini silos support the aerobic stability data. Previous studies support our results in high-moisture corn ground to achieve two different particle sizes and ensiled for up to 28 d (Saylor et al., 2020a). The increase in silage temperature is often attributed to an initial oxygen infiltration into material that may stimulate the growth of lactate-assimilating yeasts (Kung Jr. et al., 2018). Then, a subsequent pH increase can lead to exacerbated growth of molds and other undesired microorganisms, which altogether, can lead to additional heating in the silage.

### Effects of Bacterial Silage Inoculants and/or Direct-Fed Microbial Feeding on Performance and Nutrient Digestibility of Lactating Dairy Cows

#### Bacterial silage inoculants effects.

To the best of our knowledge, this is the first experiment evaluating the performance of dairy cows fed corn silage inoculated with *L. lactis* and *L. buchneri*. Therefore, any direct comparison with previous studies will not be possible and assumptions/speculations will inevitably be done during the discussion of our results. In a recent meta-analysis (*n* = 12 studies), Arriola et al. (2021) reported that feeding silage inoculated with lactic acid-producing bacteria did not impact milk yield, DMI, feed efficiency, DM digestibility, milk fat, or milk lactose, but tended to reduce milk protein content. When the sub-sample of studies feeding *L. buchneri*-inoculated corn silage was evaluated (*n* = 6), milk yield was reduced (Arriola et al., 2021). To the best of our knowledge, no other study has evaluated the effects of inoculating corn silage with *L. lactis* and *L. buchneri* on performance and nutrient digestibility of lactating dairy cows. Our results demonstrated that cows fed the inoculated corn silage had a reduced DMI (−0.5 kg/d), milk protein content (−0.03%), and somatic cell count (−0.07 log_10_), but greater milk production efficiency (+30 g milk/kg DM consumed), FPCM production efficiency (+30 g milk/kg DM consumed), and milk urea-N (+1.0 mg/dL).

Others have hypothesized that feeding an inoculated corn silage with a greater concentration of acetic acid would reduce DMI in dairy cows (Kleinschmit and Kung, 2006). However, adding 4% acetic acid to the diet of lactating dairy cows reduced DMI in the first 2 wk of the experiment, but did not impact overall DMI (Daniel et al., 2013). Therefore, it is unlikely that the content of acetic acid in the diet from our study was the main factor depressing DMI. In our experiment, cows fed the inoculated corn silage had greater total-tract apparent DM, NDF, and starch digestibility, while also tended to have a greater CP digestibility when compared with cows fed a non-inoculated corn silage. Previous studies in the literature have demonstrated that inoculating the same combination of homo- and heterofermentative bacteria as reported herein also led to a greater in situ ruminal starch degradability, indicating a potential enzymatic effect of these microbes during the ensiling process (Saylor et al., 2020a, 2020b). These results (1) support the observed reduction in DMI, as it is likely that passage rate was reduced and, in turn, (2) benefited nutrient digestibility in dairy cows, as reported by others (Weinberg et al., 2007). Nonetheless, the greater starch digestibility observed in cows fed the inoculated corn silage also led us to hypothesize that more propionate was being produced in the rumen, causing the reduction in DMI, as propionate has been linked to a reduced DMI in dairy cows (Allen et al., 2009).

The effect of feeding corn silage inoculated with a combination of *L. lactis* and *L. buchneri* on milk protein content agrees with the meta-analysis from Arriola et al. (2021). However, as reported by the authors, these results are unexpected as inoculation with *L. buchneri* and other lactic acid-producing bacteria often improves ruminal function by stimulating rumen microbes and/or microbial protein synthesis (Contreras-Govea et al., 2011). Nonetheless, one may speculate that the greater CP digestibility provided more N to the rumen than it could be used and converted into microbial protein, leading to a greater ammonia availability to reach the liver and enter the urea cycle, as more N available in the rumen leads to a greater urea-N in the blood (Cappellozza et al., 2015) and in the milk, as reported herein. In fact, Arriola et al. (2011) also reported greater blood urea-N concentration in dairy cows fed silages inoculated with *L. buchneri*.

In a meta-analysis published by Queiroz et al. (2017), milk production efficiency was increased in 25% of the studies (two out of eight) following the inoculation of the silage with heterofermentative bacteria, such as *L. buchneri*. Using grass silage, Van den Bossche et al. (2023) reported a greater milk production efficiency in dairy cows fed a grass silage inoculated with *L. buchneri* vs. hydrolyzable tannin extract. The observed improvement in milk production efficiency in our study was due to the fact that DMI was reduced, but milk yield and/or FPCM yield were maintained over the 13-wk experimental period. As mentioned before, others have reported reductions in DMI and our data also support this finding, but the maintenance of milk yield and resulting improvement in milk production efficiency is a novel finding of the combination of *L. lactis* and *L. buchneri* inoculated in corn silage. In fact, the greater nutrient digestibility reported herein might have compensated for the reduction in DMI, supplying the nutrients required for the maintenance of milk production in high-producing dairy cows. In lambs, gain to feed ratio did not differ, but average daily gain corrected for carcass weight was greater when corn silage was inoculated with *L. plantarum* and *L. buchneri*, even though nutrient digestibility was lower (Basso et al., 2014)

#### Direct-fed microbial effects.

Previous studies conducted with a similar DFM combination as evaluated herein reported greater milk yield in transition dairy cows, *in sacco* forage nutrient digestibility, and a greater mean rumen pH of dairy cows following a subacute rumen acidosis challenge (Nocek et al., 2002, 2003; Nocek and Kautz, 2006). In the present study, we observed greater milk yield, FPCM yield, greater protein yield, and lactose yield, but reduced milk fat and protein content, without changes in nutrient digestibility. In transition dairy cows, Nocek et al. (2003) reported that feeding *E. faecium* and *S. cerevisiae* pre- and post-calving improved milk production and milk protein content. Moreover, in a subsequent study by the same research group (Nocek and Kautz, 2006), milk yield was greater for post-partum dairy cows fed *E. faecium* and *S. cerevisiae*, no differences were observed on 3.5% fat-corrected milk yield and milk protein content, but milk fat content was reduced in DFM-fed cows. As no treatment differences were observed on DMI and apparent nutrient digestibility, it may be speculated that the main mode of action supporting and explaining these results relies on tonic and steady production of lactic acid production in the rumen, which is used by specific lactic acid-utilizing bacteria and/or yeast (Dawson, 1995). The result would be the production of sustained low and nondetectable concentrations of lactic acid in the rumen, supporting a basal level of lactic acid utilizers, which would tend to stabilize and increase pH, particularly nadir levels (Owens et al., 1998; Nocek et al., 2002). Differences among studies and some of these results might be related to the nutritional characteristics of the diet and production stage of the herd (Seo et al., 2010; McAllister et al., 2011). It is important to recognize that all the aforementioned studies did not add Torula yeast to the formulation of the DFM. Torula yeast has been categorized as a nutritional yeast when fed as an inactive microbial biomass for nutritional value (Shurson, 2018). It has the ability to metabolize xylose and xylose oligomers (Yanai and Sato, 2001), representing an opportunity to produce large amounts of microbial protein from a sustainable and cost-effective growth medium (Holt and Aldrich, 2022).

Other authors have also reported changes in milk composition when lactating dairy cows were fed DFM (McGilliard and Stallings, 1998). As there were no differences in milk fat yield, the significant difference observed in milk fat content can be attributed to a dilution effect of the greater milk production in DFM-fed cows. The improvements in milk protein and lactose yields following DFM feeding could be related to the commensal effects of probiotics in the rumen environment and microbiota, stimulating the growth of beneficial microorganisms that enhanced the total amount of microbial protein produced and absorbed in the lower GIT (Nalla et al., 2022).

Current challenges in ruminant production systems include increased feed and operational costs and it has been recognized that feed costs represent the biggest threat to the profitability of a dairy operation (Bach, 2012). Therefore, improving and/or maximizing milk production efficiency could be a way to directly benefit the profitability of dairy operations (Connor, 2015). Supporting our results, Oyebade et al. (2023) reported that cows fed a combination of different bacterial DFM strains had greater energy-corrected milk production efficiency when compared with non-supplemented cows. Valldecabres et al. (2022) also showed improvements in milk production efficiency in lactating dairy cows fed a four-strain vs. two-strain DFM or non-supplemented cows throughout lactation. Therefore, it can be speculated that the improvements in milk production efficiency are likely to be multi-factorial, including supportive effects in the rumen and the lower GIT.

#### Bacterial silage inoculants × DFM.

 Adding DFM to the diet of animals fed a bacterial-inoculated corn silage improved milk production efficiency when compared with CON-CON but did not differ from cows fed an inoculated corn silage only. On the other hand, feeding a bacterial-inoculated corn silage with DFM reduced SCC when compared with all other treatments. Somatic cell counts in the milk have been considered a biomarker of mammary gland health and inflammation for intramammary infections (i.e., mastitis; Schukken et al., 2003), so that reduced counts may reflect a lower risk for mastitis (Williamson and Lacy-Hulbert, 2013) and an effective mastitis management practice in the dairy cow herd (Lago et al., 2016). Alawneh et al. (2020) demonstrated that using *Lactobacillus* spp. in a teat udder spray tended to reduce the somatic cell counts in dairy cows. *Bacillus subtilis* feeding significantly decreased the incidence of mastitis, the average number of medication days and the number of days that the milk was discarded, and the somatic cell count in cows between 51 and 75 d post-calving when compared with the values of cows in the previous lactation (Urakawa et al., 2022). Cattaneo et al. (2023) reported that transition dairy cows fed *S. cerevisiae* had a lower somatic cell count 28 d post-calving, suggesting that DFM might benefit mammary gland and overall health of the herd. These studies hypothesized that formation of biofilm, stimulus of growth and development of commensal bacteria in the mammary gland, as well as direct inhibitory effects against pathogens could be playing a role. However, considering the stage of lactation of the cows enrolled in our study, direct comparisons with the aforementioned studies may not be fully accurate.

The reduction in milk protein content was more pronounced in cows fed both inoculated corn silage and DFM, corroborating with the main effects of the factors (silage inoculant and DFM). The apparent explanation, if any, for the results reported here is not clear at this point but may involve a greater CP digestibility and an increased biomass protein production in the rumen that ultimately might impact the supply of microbial protein to the mammary gland and, therefore, to the milk of the cows. However, additional studies are warranted to understand the effects of bacterial silage inoculants and DFM supplementation on rumen function, metabolism, and performance responses of lactating dairy cows.

## Conclusions

In summary, our study supports our hypothesis that inoculating a combination of *L. lactis* and *Lentilactobacillus buchneri* improved acetic acid content and increased aerobic stability of corn silage, and also improved milk production efficiency and nutrient digestibility in lactating Holstein dairy cows. On the other hand, feeding a direct-fed microbial containing *Enterococcus faecium*, *Saccharomyces cerevisiae*, and Torula improved milk yield, as well as protein and lactose yield. Nonetheless, combining the feeding of an inoculated corn silage with DFM reduced somatic cell count in the milk. Additional research efforts are warranted to understand how different bacterial DFM species and/or strains interact with inoculated silages to improve performance, health, and nutrient digestibility of lactating dairy cows.
